# Sources of QTc variability: Implications for effective ECG monitoring in clinical practice

**DOI:** 10.1111/anec.12730

**Published:** 2019-11-24

**Authors:** Katerina Hnatkova, Marek Malik

**Affiliations:** ^1^ National Heart and Lung Institute Imperial College London UK

**Keywords:** QT/RR hysteresis, QT heart rate correction, QT measurement, Serial QTc monitoring, T‐wave morphology

## Abstract

Pharmaceuticals that prolong ventricular repolarization may be proarrhythmic in susceptible patients. While this fact is well recognized, schemes for sequential QTc interval monitoring in patients receiving QT‐prolonging drugs are frequently overlooked or, if implemented, underutilized in clinical practice. There are several reasons for this gap in day‐to‐day clinical practice. One of these is the perception that serially measured QTc intervals are subject to substantial variability that hampers the distinction between potential proarrhythmic signs and other sources of QTc variability. This review shows that substantial part of the QTc variability can be avoided if more accurate methodology for electrocardiogram collection, measurement, and interpretation is used. Four aspects of such a methodology are discussed. First, advanced methods for QT interval measurement are proposed including suggestion of multilead measurements in problematic recordings such as those in atrial fibrillation patients. Second, serial comparisons of T‐wave morphologies are advocated instead of simple acceptance of historical QTc measurements. Third, the necessity of understanding the pitfalls of heart rate correction is stressed including the necessity of avoiding the Bazett correction in cases of using QTc values for clinical decisions. Finally, the frequently overlooked problem of QT‐heart rate hysteresis is discussed including the possibility of gross QTc errors when correcting the QT interval for simultaneously measured short‐term heart rate.

## 1 INTRODUCTION

Number of pharmaceuticals that are used in both hospital‐based and ambulatory care can cause QT prolongation with the danger of life‐threatening arrhythmias in susceptible patients (Al‐Khatib et al., 2018; Drew et al., 2010). This proarrhythmia danger in susceptible patients (Vandael, Vandenberk, Vandenberghe, Willems, & Foulon, 2017) occurs not only with antiarrhythmic treatment but also with many other compound classes, including fluoroquinolones and other antibiotics, antipsychotics, anticancer drugs, immunosuppressants, monoclonal antibodies, and others. The regulatory agencies therefore postulate that the use of some of these drugs mandates serial QTc evaluations based on initiation and/or maintenance electrocardiogram (ECG) monitoring. Indeed, different healthcare providers stipulate guidelines and schemes for such a monitoring (HERPC, 2019). Scoring systems suggesting probability of QTc prolongation have also been reported (Tisdale et al., 2013).

The effectiveness of the monitoring schemes and their practical clinical implications have been the topic of numerous surveys, metanalyses, and reviews (Pezo, Yan, Earle, & Chan, 2019; Sharma et al., 2017; Warnier et al., 2015). These lead to the conclusion that the monitoring schemes result in an increase in the knowledge and awareness of the drug‐induced QTc prolongation with consequent proarrhythmic risk among the clinical community. At the same time, however, the available literature also suggests that in terms of clinical implications, for example, therapy changes in susceptible patients, the ECG monitoring schemes are frequently not particularly successful (Good, Riad, Good, & Shalaby, 2016).

There are number of reasons for these methodological failures. As well known, the duration of the QTc interval is influenced by plasma electrolytes (Facchini et al., 2006; Genovesi et al., 2008, 2019) that might easily change during the treatment course. QTc is also influenced by fever (Drew, Baranchuk, Hopman, & Brison, 2017) and many other conditions including central nervous (Capparelli et al., 2013) and hormonal changes (Albert, Eckersley, Skinner, & Jefferies, 2014). All this leads to variability in the sequentially measured QTc values that is not only challenging to control for but also makes it difficult to differentiate between the truly proarrhythmic signs and other influences of the repolarization control. Considering this multifactorial QTc variability, it is not too surprising that the value of clinical QTc monitoring might be questioned (Benjamin et al., 2018).

Nevertheless, considering the standard practices of recording, displaying, and measuring ECG recordings, it is also apparent that the QTc variability and thus the problems with QTc monitoring might be “man‐made.” This has recently been well documented by Gueta et al. (2019) who demonstrated that using the usual standard evaluation approaches, serial ECG recordings obtained over prolonged periods of time show fairly variable QTc readings even in healthy individuals free off any QT‐related treatments or procedures. In their study, Gueta et al observed serial QTc changes commonly exceeding limits that have previously been proposed to signify substantial repolarization changes and that are considered to mandate drug withdrawal of other treatment changes in clinical cases subject to serial ECG monitoring.

There are two interpretation facets to this observation. On the one hand, one could, similar to Gueta et al, ascribe the serial QTc differences to within‐individual variability. This might potentially lead to a bleak conclusion that QTc intervals derived from standard clinical ECGs should not be used as a validated sign of adverse drug effects leading to withdrawal of potentially important treatments. On the other hand, however, one could critically review the usual standards of clinical electrocardiography, well reflected in the study by Gueta et al, and consider possible sources of QTc variability that are more or less methodological and completely independent of or at least largely remote from the true biological within‐subject QTc variations.

In this text, we aim at reviewing and discussing four facets of ECG measurement and QTc comparisons that might mitigate the undesirable consequences of methodology‐induced QTc variability.

## 2 ELECTROCARDIOGRAPHIC MEASUREMENTS

Consistent with prevailing clinical practice, Guetta et al used standard printout of 12‐lead ECGs (25 mm/s paper speed, 10 mm/mV amplitude) and measured QT interval duration in lead II. They made the measurements manually with a ruler allowing 0.5‐mm resolution. Nevertheless, these standard approaches also appear to be a part of the variability problem.

**Figure 1:**
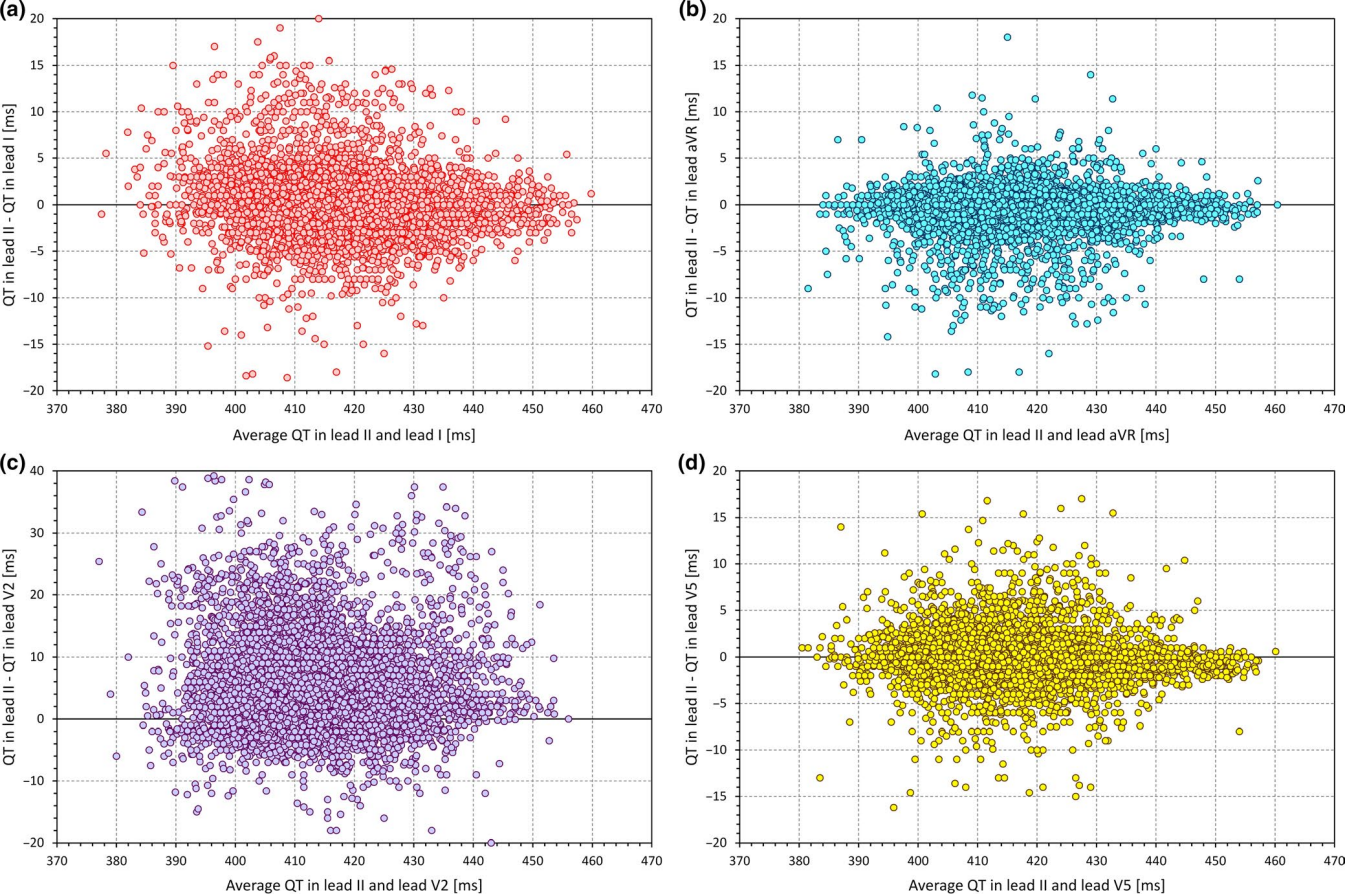
QT interval measurements in 8,225 digital 12‐lead ECGs obtained in healthy subjects: In each of the ECGs, QT intervals were measured (where measurable) in each lead by at least two independently working cardiologists which subsequent reconciliation of their differences. The figure shows the comparisons of QT interval durations measured in lead II with the measurements in other leads. Panels a, b, c, and d show scatter diagrams of the QT differences between leads II and leads I, aVR, V2, and V5, respectively, against the averages of the measurements in lead II and in the respective leads

As well known, the interlead discrepancies in the QT interval measurement are caused, apart from measurement inaccuracies, mainly by the different projection of the spatial T‐wave loop into the different ECG leads (Kors & van Herpen, 1998; Kors, Herpen, & Bemmel, 1999; Lee, Kligfield, Dower, & Okin, 2001). Frequently, lead II is considered to contain the longest, and thus the most representative QT interval duration. (The frequent selection of lead II for QT measurement is possibly also influenced by the simple technical fact that in standard ECG displays, most electrocardiographic machines show the full rhythm strip in lead II.) Such an assumption is not supported by data. For instance, Figure 1 shows analysis of more than 8,000 digital ECGs recorded in healthy subjects in supine positions. In each of these ECGs, the QT interval duration was measured by two or more independently working cardiologists, with averaging five different measurements of the given lead and with reconciliation of disagreements between the observers. The QT interval duration in lead II was the longest of all leads only in some 11% of the cases and its difference from the true maximum reached 50 ms in some cases. This does not mean that other singular ECG leads would be clearly preferable to lead II. As seen in Figure 1, similar inconsistencies also exist with measurements made in other leads. This is not surprising, since the T‐wave lead projection depends on the actual orientation of the spatial T‐wave loop. This is not only individual but also dependent on position of the heart in the thorax which, in turn, depends not only on body position but also on meal ingestion and many other factors. Thus, in daily practice, when the QT interval duration determines important clinical decisions, single lead measurements should not be relied on. Assurance of the validity of the measurement comes from the mutual correspondence between different ECG leads.

Consistent with standard metrology principles (Squara, Imhoff, & Cecconi, 2015), Guetta et al also correctly emphasized the importance of averaging multiple measurements. Unfortunately, it is questionable whether averaging of multiple measurements (of both QT and RR intervals) is regularly used in standard clinical ECG measurements. If no averaging is implemented and if only one beat is considered, it is not surprising that the validity and accuracy of the QTc reading are very low.

**Figure 2:**
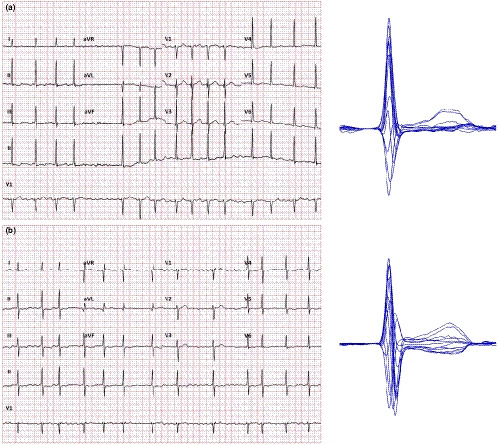
Digital 12‐lead ECGs recorded in patients with atrial fibrillation (a—67‐year‐old female, b—70‐year‐old male). In both cases, any reasonably accurate QT interval measurement is clearly difficult if not impossible in the standard 12‐lead printouts shown on the left‐hand side. (Note that while T waves are visible in some of the precordial leads of both tracings, their morphology in individual beats is influenced by atrial fibrillatory waves which make the QT interval measurement inconsistent between individual cycles.) On the right‐hand side, corresponding representative complexes are shown constructed with automatic determination of the QRS onset and superimposition of the segments preceding the QRS complex in all leads. While some noise due to the fibrillatory waves is still visible in the representative beatforms, QT interval duration can be made with sufficient confidence in both cases

For ECG processing, this does not necessarily only mean averaging the separate measurements of the same lead in multiple QRS‐T complexes. The averaging process can also be applied to the signals of properly aligned individual complexes to obtain the so‐called representative QRS‐T beatforms. When aligning the individual complexes and using sample‐by‐sample voltage medians rather than sample‐by‐sample averages, the process also filters the native recording and creates images that are easier to interpret. This is also true for recordings in which the QT interval measurement in individual beats is problematic because of underlying biological noise (Figure 2), such as ECGs of patients with atrial fibrillation (Tooley et al., 2019) or in Parkinson's disease patients (Malik, Andreas, et al., 2008a). The only exception in which this technology fails is fixed ratio atrial flutter with phase‐locked flutter waves and QRS complexes and with superimposition of flutter waves with the terminal part of the T wave.

While such representative beatforms are usually not included in standard ECG displays (perhaps apart from exercise and other specific recordings), many manufacturers of digital ECG equipment offer tools for their construction. When the representative beatforms of individual leads are displayed on the same isoelectric axis, comparison of the QT duration in different leads is also possible further increasing the accuracy of the measurement. Measuring the QT interval in this so‐called butterfly plot (Malik, 2004) clearly does not belong to the day‐to‐day clinical practice. Nevertheless, it can only be advocated in cases when the QT interval duration is used for important therapeutic decisions.

## 3 CONSISTENCY OF MORPHOLOGICAL INTERPRETATIONS

Manual measurement of paper printed ECGs also needs to consider, among others, the width of the line of the printed tracing. This can easily be around 0.5 mm which, with the standard paper speed, corresponds to 20 ms. It is thus questionable whether manual measurements should be used on their own or whether it is more reliable to visually check the automatic measurements provided by most of the advanced models of ECG equipment (Hnatkova, Gang, Batchvarov, & Malik, 2006). Similar to the necessity of having serial ECGs interpreted by the same observer, ECG measurements by different equipment may lead to substantial variability (Kligfield et al., 2018, 2014). However, since the algorithms used in the commercial equipment are steadily advancing (Green et al., 2012) their clinical reliability is now probably at least equivalent to fully manual measurements especially if combined with visual verifications to eliminate occasional outliers (Hnatkova et al., 2006).

While the identification of the QRS complex onset might occasionally be highly problematic, the difficulty of QT interval measurement stems mainly from the identification of the T‐wave offset. The gradual transition of the downslope of the T wave into the isoelectric line or, perhaps more frequently, into the subsequent U wave makes any definition of the T‐wave end highly dependent on the perception and interpretation of the ECG patterns. Unfortunately, human readers are also not particularly accurate in maintaining the ECG interpretation constant and in measuring similarly shaped T waves consistently (Johannesen, Garnett, & Malik, 2014a, 2014b). The inaccuracies caused by this inability of human observers to maintain the same interpretation approach to different ECGs might only occasionally be substantial enough to trigger undesirable treatment consequences. Nevertheless, the existence of these inaccuracies calls for both the help provided by automatic algorithms that suffer much less from the “systematicity” problem and for the serial comparison and reinterpretation of ECG tracings rather than only blind evaluations of historical QT/QTc readings.

Many hospital information systems store only the images of recorded ECGs rather than electronic data of individual voltage values. This is unfortunate since simple images do not allow the morphologies of serial recordings to be easily compared, for example, by overlay of T‐wave morphologies, which clearly increases the precision of serial evaluations. Advanced systems for ECG storage thus need to be advocated (Sassi et al., 2017).

## 4 HEART RATE CORRECTION

Consistent with previous criticism of the Bazett formula (Indik, Pearson, Fried, & Woosley, 2006; Malik, 1996; Rautaharju, Warren, & Calhoun, 1990) Guetta et al found that with Bazett correction, the incidence of substantial QTc changes was much larger compared with other correction formulas. This is not surprising since compared with many other correction formulas, Bazett correction is more influenced by the underlying heart rate changes (Hnatkova, Vicente, Johannesen, Garnett, Stockbridge, et al., 2019a). Indeed, Guetta et al found the heart rate differences between serial ECG to be much greater in subjects who showed substantial QTc changes compared with those who did not. It seems only little odd to group the correction errors and computational artifacts under the label of within‐subject variability.

Understandably, heart rate differences between serial clinical ECGs cannot easily be eliminated. This, combined with the long‐known problems of Bazett formula, led to a multitude of proposals of other correction formulas none of which solved the problem satisfactorily. More than two decades ago, we have also fallen into the trap of believing that it is sufficient to accumulate ECGs from a large number of individuals to describe a valid physiologic QT‐heart rate relationship with sufficient accuracy and that a valid physiologic relationship can be converted into an optimum correction formula (Hnatkova & Malik, 1999). Only subsequently, it became obvious that all the attempts to develop a universally applicable formula are fruitless and nonsensical since there is no physiologically valid QT‐heart rate relationship applicable to all or most individuals (Batchvarov et al., 2002; Malik, Färbom, Batchvarov, Hnatkova, & Camm, 2002).

**Figure 3:**
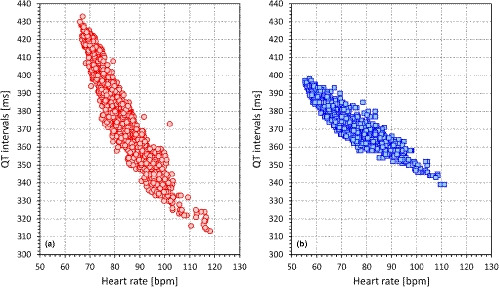
Example of QT/heart rate profiles obtained in a 31‐year‐old healthy female (panel a) and a 46‐year‐old healthy male (panel b). Each profile is based on more than 1,000 measurements that were made during a span of approximately 1 month. The heart rate values on the horizontal axes were individually corrected for QT/RR hysteresis (Malik, Hnatkova, Novotny, et al., 2008b)

The observation that the QT‐heart rate relationship differs between different individuals as much as the papillary lines of their fingerprints have profound implications for serial comparisons of QTc intervals. The problem is highlighted in Figure 3 which shows the QT‐heart rate relationship free of any drugs or other nonphysiologic influences in two healthy subjects. The Figure shows that if changing the heart rate from 70 to 100 beats per min, the QT interval changes by 30 ms in one of the subjects and by 80 ms in the other. This makes it obvious that there cannot possibly be a correction formula that would reasonably work in both cases. More importantly, without a detailed investigation, it is impossible to estimate the QT‐heart rate profile in a given patient (i.e., it is impossible to guess whether a QT‐heart rate profile of a given patient is closer to case A or to case B in Figure 3). Hence, if ECGs before and after treatment are compared and if they noticeably differ in heart rate, it is practically impossible to say whether the QT interval was changed by the drug above or below the level that could be attributed to the heart rate change in the given patient (Malik et al., 2019).

Recently, we reported that for regulatory investigations of drug‐induced QTc interval changes, Framingham or Fridericia formulas may be reasonably used if the underlying heart rate was not changed by more than 10 beats per minute (Hnatkova, Vicente, Johannesen, Garnett, Stockbridge, et al., 2019a). In clinical practice, larger errors of QTc assessments may be accepted. Nevertheless, the same experiments still suggest that with heart rate changes in excess of 15 or possibly 20 beats per minute, no fixed correction formula can be relied on for the purposes of clinical decisions. Referring again to the study by Guetta et al, it would be interesting to know how many QTc excesses were found with Fridericia or Framingham corrections if the heart rate differences did not exceed 15 beats per minute.

## 5 HEART RATE HYSTERESIS

While the inaccuracies of heart rate correction formulas are well‐known and largely understood, albeit recurrently neglected in clinical practice, little attention is paid to the potentially substantial errors in QTc intervals due to incorrect heart rate measurements. It has been repeatedly described that QT interval duration does not depend on (and thus should not be corrected for) instantaneously measured heart rate but that it responds to heart rate instability with a considerable delay (Gravel, Jacquemet, Dahdah, & Curnier, 2018; Malik, Hnatkova, Novotny, & Schmidt, 2008b; Pueyo, Smetana, Laguna, & Malik, 2003). However, this so‐called QT/RR hysteresis is regularly completely ignored in clinical practice (e.g., Guetta et al do not mention the problem in their report and it is not obvious whether they considered it.). At the same time, QT interval adaptation takes much longer than the 10‐s duration of standard electrocardiograms and thus, room for very substantial errors exists (Garnett et al., 2012).

**Figure 4:**
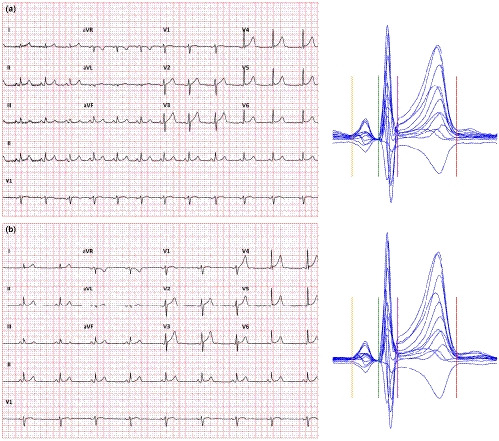
Digital 12‐lead ECGs recorded in a 45‐year‐old healthy male off any medication. The recording a shown on the top started 20 s before the recording B shown on the bottom. The averaged 10‐s heart rates were 73.5 and 53.4 beats per min in recordings a and b, respectively. The images of representative beats of all 12 leads superimposed on the same isoelectric axis are shown on the right side of each panel. These also show the measurement triggers of P onset (amber line), QRS onset (green line), J point (violet line), and T offset (red line). The uncorrected QT interval in both tracings was the same (428 ms). When using the 10‐s heart rate and correcting the QT interval by Bazett correction, QTc intervals of 474 and 403 ms were obtained. With Fridericia correction, the QTc values were 458 and 411 ms. When using individual correction that also involved individual QT/RR hysteresis component, QTc values of 417 and 419 ms were obtained

Example of the problem is shown in Figure 4 which shows two 10‐s ECGs recorded in a healthy subject who was in a strict supine position for more than 5 min prior to the first ECG. These two tracings were separated by only a 10‐s gap between them and still, their heart rate differed by more than 20 beats per min. The figure also shows that the uncorrected QT interval was the same as the time that elapsed between the two recordings was too short for the QT interval to adapt to the new or transient heart rate levels. When the QT interval was corrected for instantaneously measured 10‐s heart rate, Bazett and Fridericia correction showed difference of 73 and 47 ms, respectively. However, when the 5‐min heart rate history (also available in this case) was used for individual QT/RR hysteresis correction (Malik, Hnatkova, Novotny, et al., 2008b), the corrected QTc intervals differed by only 2 ms.

Correcting for QT/RR hysteresis in clinical practice is clearly beyond usual practical day‐to‐day possibilities (Hnatkova, Vicente, Johannesen, Garnett, Strauss, et al., 2019b; Malik, Johannesen, Hnatkova, & Stockbridge, 2016). Nevertheless, serious attention needs to be given to the phenomenon. Although physical reasons for heart rate differences can be eliminated by maintaining undisturbed position for a sufficiently long period before ECG recording, psychological and mental reasons for heart rate fluctuations are completely beyond clinical control. Indeed, we have previously reported that in clinical pharmacology studies, heart rate differences between closely coupled ECGs were frequently much larger compared with the example in Figure 4 although the investigated subjects were, per protocol, kept in supine resting positions for several minutes before the first ECG was recorded (Malik et al., 2016).

It can thus only be recommended that when valid QTc duration is needed for important clinical decisions, several closely coupled serial ECGs are recorded in order to ascertain the stability of heart rate preceding the recording in which the QT interval is measured. If QT interval is recorded and corrected for simultaneously measured heart rate and if the heart rate was not stable in the preceding minutes, the disparity between the measured heart rate and the heart rate that influences the QT interval duration may lead to QTc errors. These errors (caused by correcting the QT interval for a “wrong” heart rate) may be very substantial and can easily be larger than the errors caused by an inappropriate heart rate correction formula (Hnatkova, Vicente, Johannesen, Garnett, Stockbridge, et al., 2019a).

## 6 CONCLUSION

QTc interval monitoring plays an important role in clinical decisions of therapy maintenance when using drugs with known proarrhythmic potential. Nevertheless, casual clinical practice may result in substantial QTc variability which might easily compromise if not invalidate serial ECG monitoring schemes. Naturally, disease progression and aging linked to clinical or subclinical heart disease may impact on valid QTc values. Nevertheless, in healthy subjects, the QTc interval is fairly stable not only over short time spans as repeatedly reported (Malik, Hnatkova, Schmidt, & Smetana, 2008c) but also over years (Batchvarov et al., 2000). The QTc variability seen in clinical practice can (and should) thus be substantially reduced if paying attention to measurement and interpretation details. It appears that major problem is related to the use of improper heart rate corrections. As repeatedly reported, not only Bazett correction should be replaced by more accurate corrections (e.g., Fridericia or Framingham) but also, and perhaps more importantly, the dire problems of comparing QTc values measured under very different heart rates should not be forgotten. If using a drug with a known proarrhythmic potential combined with expected large heart rate effects, patient‐specific QT/heart rate profile should first be established so that subsequent safety monitoring can separate the drug adverse effects from the heart rate adaptation.
